# Integrating genomic additive relationship matrices improves the efficiency in diploid banana breeding

**DOI:** 10.1093/hr/uhag139

**Published:** 2026-04-14

**Authors:** Violet Akech, Rony Swennen, Therése Bengtsson, Rodomiro Ortiz, Stanley Bayo, Marnik Vuylsteke, Brigitte Uwimana, Allan Brown

**Affiliations:** Crop Improvement Department, International Institute of Tropical Agriculture, Kampala Uganda; Department of Plant Breeding, Swedish University of Agricultural Sciences, Alnarp, Sweden; Crop Improvement Department, International Institute of Tropical Agriculture, Kampala Uganda; Department of Biosystems, KU Leuven, Willem De Croylaan 42, B 3001 Heverlee, Belgium; Department of Plant Breeding, Swedish University of Agricultural Sciences, Alnarp, Sweden; Department of Plant Breeding, Swedish University of Agricultural Sciences, Alnarp, Sweden; Crop Improvement Department, International Institute of Tropical Agriculture, Arusha, Tanzania; Gnomixx B.V., Melle, Belgium; Crop Improvement Department, International Institute of Tropical Agriculture, Kampala Uganda; Crop Improvement Department, International Institute of Tropical Agriculture, Arusha, Tanzania

## Abstract

Partitioning of genetic variance into additive and non-additive components using the pedigree-based best linear unbiased prediction (P-BLUP) model is possible because of the family structure and replicated clones in clonally propagated crops, but this model may overestimate these components. However, the genomic best linear unbiased prediction (G-BLUP) method, which integrates the genetic relationship through molecular marker information reduces the overestimation. Alternatively, a combination of the P-BLUP and G-BLUP, sourcing to create a hybrid matrix that estimates hybrid best linear unbiased prediction (H-BLUP), is proposed. We investigated if integrating molecular information into the clonal model could improve the partitioning of the variance components leading to more accurate estimates of genetic parameters and prediction accuracy of breeding values of 14 key traits in diploid banana. In this study, we used clones of 14 full-sib families from a factorial mating design of four female and five diploid male banana (*Musa acuminata*) parents, generated at the International Institute of Tropical Agriculture in Arusha. The genomic-based relationship matrices were constructed using a set of 2792 filtered single-nucleotide polymorphism markers. Additive variance and heritability derived from G-BLUP and H-BLUP models reduced bias compared to the P-BLUP model. The H-BLUP estimated the highest prediction accuracies for yield-related and cycling traits, while the P-BLUP model had the highest prediction accuracy estimates for agronomic traits. The use of marker-based models enhances the accuracy of predicting breeding values, contributing to accurate estimates of genetic gain while paving a way for further genomic exploration in diploid banana breeding programs.

## Introduction

Grown in over 130 countries by both small- and large-scale farmers, bananas (*Musa* spp.) are the most popular fruits worldwide, with an estimated global production ranging from 114 to 139 million tons per year [1, 2]. Banana is a staple for over 30 million people in the Great Lakes Region of East Africa (that includes Burundi, Rwanda, eastern Democratic Republic of the Congo, Uganda, and northwestern Kenya and Tanzania) [3, 4]. Matooke (AAA subgroup) and Mchare (AA subgroup) bananas that are cooked before consumption [5] dominate production of cooking bananas in Uganda and Tanzania. They represent a unique set of *Musa* germplasm mainly found in East and part of Central Africa region [6–8].

Despite its importance and high-volume demands, current yields in East Africa are still low compared to potential yields of 70 t∙ha^−1^∙year^−1^ [9–11]. Developing disease and pest-resistant, high-yielding cultivars with acceptable quality are the primary objectives of banana breeding programs [12]. The type of banana grown, whether local cultivars or hybrids, is among the prevailing factors that account for yield differences among farmer's fields [10, 13, 14]. Genetic improvement has proven to be the most sustainable approach to increase yield by developing improved hybrids showing stable high yields, resilience to biotic and abiotic stresses, and that meet consumers' quality preferences. A better understanding of the genetic values of the parents and breeding clones used in banana improvement programs is essential in making decisions regarding breeding strategies if breeding efficiency and improved genetic gains are to be realized.

Improved triploid banana hybrids have mainly been developed through crossbreeding which follows the classical process that first focuses on the improvement of the diploid (2*x*) parents from the wild genotypes to eliminate the non-domestication traits, then the improved 2*x* are crossed with triploid (3*x*) landraces, to generate (primarily) tetraploids (4*x*). The tetraploids are further crossed with an improved diploid to generate secondary 3*x* hybrids that are parthenocarpic, high yielding, resistant to pests and diseases, and have fruit qualities comparable to those of the original landraces [12, 15, 16]. This method has been adopted by the few existing banana breeding programs to develop cultivars [17]; [8, 18, 19]).

Crossbreeding begins with selecting suitable parental lines that possess the desired characteristics for crossing. The choice of parents for crossing determines the efficiency of combining genes in plant breeding and the genetic variability among progeny populations, which eventually is exploited during selection [20]. Prediction of crosses is hindered due to limited knowledge concerning the genetic potential of parental clones and the genetic architecture of essential traits. Genetic gains can be increased by making crosses with genotypes that complement each other, due to accumulation of additive genes [21, 22]. Parent selection is based on the genotype's potential to produce high proportions of progenies with high trait values [16].

The challenge with improving East African highland bananas is attributed to, among other factors, its domestication where selection for desirable traits, such as seedlessness and larger fruit size led to dominance of parthenocarpy and polyploid nature of the crop [23], leading to reduced fertility and hence poor seed set [24, 25]. Reduced fertility limits the number of hybrids that can be obtained for evaluation and selection. Banana is a long-cycle crop that requires usually two to three years of field evaluation, which makes the identification of good parents with superior combining ability slow, expensive and tedious [24]. In addition, a proportion of the plants are lost during the extended growth cycle, resulting in incomplete data collection. While the perennial nature of banana enables year-round harvests and consumption, ensuring food and income security [12, 26], it poses a challenge for breeders during the evaluation and selection of hybrids. A typical banana evaluation field consists of a population of individual plants that are at different developmental stages, at any given time [27] leading to unsynchronized data. All these challenges in banana breeding reduce the response to selection [28].

Understanding the genetic variation available in the diploid banana (Mchare) breeding program simplifies the analysis by removing complications associated with triploid programs and is essential to leverage the variants for parental improvement and to obtain genetic gains [29]. Partitioning the genetic variation into additive, dominance, and epistasis components, helps to avoid the inflation and the confounded estimates of the genetic parameters, such as narrow sense heritability, especially in complex traits that are governed by many genes with small effects and their interactions [30–33]. In addition, determining how much of the genetic variation is due to additive, dominance, and epistasis effects improves the estimation of the breeding values of individuals under investigation [34–36]. This aids the breeder to select the improvement strategy for the traits of interest, for example, whether to focus the breeding efforts and resources on parent selection, family selection, within-family (clones) selection or a combination of approaches.

The full clonal model [33, 37, 38], an extension of the animal model, incorporates relationship information into a linear mixed model (LMM) to partition the genetic variance of the trait of interest into additive [i.e. estimated breeding values (EBVs)], dominance, and epistasis effects. This is possible because of the family structure and replicated clones in clonally propagated crops as demonstrated in Norway spruce (*Picea abies*) [37], in loblolly pine (*Pinus taeda*) [33], in sugarcane [38], and in banana [39]. The clonal model, like the animal model, can deal with complex and unbalanced datasets [40], which is often experienced in clonal and perennial crops, such as banana. One method of estimating the genetic effects is known as pedigree-based best linear unbiased prediction (P-BLUP) [41, 42], where the genetic co-ancestry information is calculated based on the expected coefficient of relationship between any pair of individuals [30, 43]. Equivalently, genetic parameters of interest can be estimated by the genomic best linear unbiased prediction (G-BLUP) method [44], which integrates the genetic relationship established through molecular information. In this case, dense genome-wide marker data is used to estimate a realized relationship matrix (G Matrix) based on the observed proportion of the alleles that are shared by each pair of individuals [45–47]. G-BLUP accounts for variation in expected identical-by-descent (IBD) values arising from Mendelian sampling during gametogenesis by estimating the real proportion of IBD alleles [43, 48]. Alternatively, a combination of the P-BLUP and G-BLUP information can be used as a hybrid genomic relationship matrix (H Matrix) through the single-step GBLUP (ssGBLUP) methodology, which combines the A matrix and the G matrix to give estimates of hybrid best linear unbiased prediction (H-BLUP) [49]. The first study to apply the clonal model for handling complex and unbalanced dataset in banana hybrid evaluation primarily focused on the use of the P-BLUP method [50]. Our study builds on this work by applying P-BLUP to a new and larger population, while also implementing the H-BLUP approach to improve model accuracy in partitioning variance components, estimating narrow-sense heritability, and predicting genetic values for key traits. The H-BLUP method enables the inclusion of both genotyped-only and phenotyped-only individuals, thereby increasing the effective number of observations. Moreover, it allows the model to leverage genomic relationships to account for plants or phenotypes lost during the extended evaluation period, a common challenge in banana breeding trials.

In this study, we investigated whether integrating genomic information into the full-clonal model will improve the partitioning of the variance components, yielding more accurate estimates of heritability and genetic effects to improve efficiency in diploid banana breeding. To achieve this, genetic marker information was used to construct an additive matrix (G-BLUP), replacing the pedigree-based additive matrix (P-BLUP) or combining it with a pedigree-based additive matrix to estimate H-BLUP.

## Results

### Quality and diversity of the markers

A sum of 14 587 unprocessed SNP markers was generated from the DArTseq analysis of the banana hybrids and the nine parental varieties. Following the filtering procedure, 2792 SNPs (19%) and 68 genotypes (92%) met the quality standards required and were retained for the construction of the genomic relationship matrix. Table 1 shows the characteristics of the retained 2792 SNP markers.

**Table 1:** Profile of the SNP markers used to construct the genetic relationship matrix.

Parameters	Min	Max	Mean
Observed heterozygosity	0.00	0.25	0.14
Expected heterozygosity	0.10	0.50	0.29
Polymorphic information content	0.09	0.50	0.28
MAF	0.05	0.50	0.21

**Figure 1:**
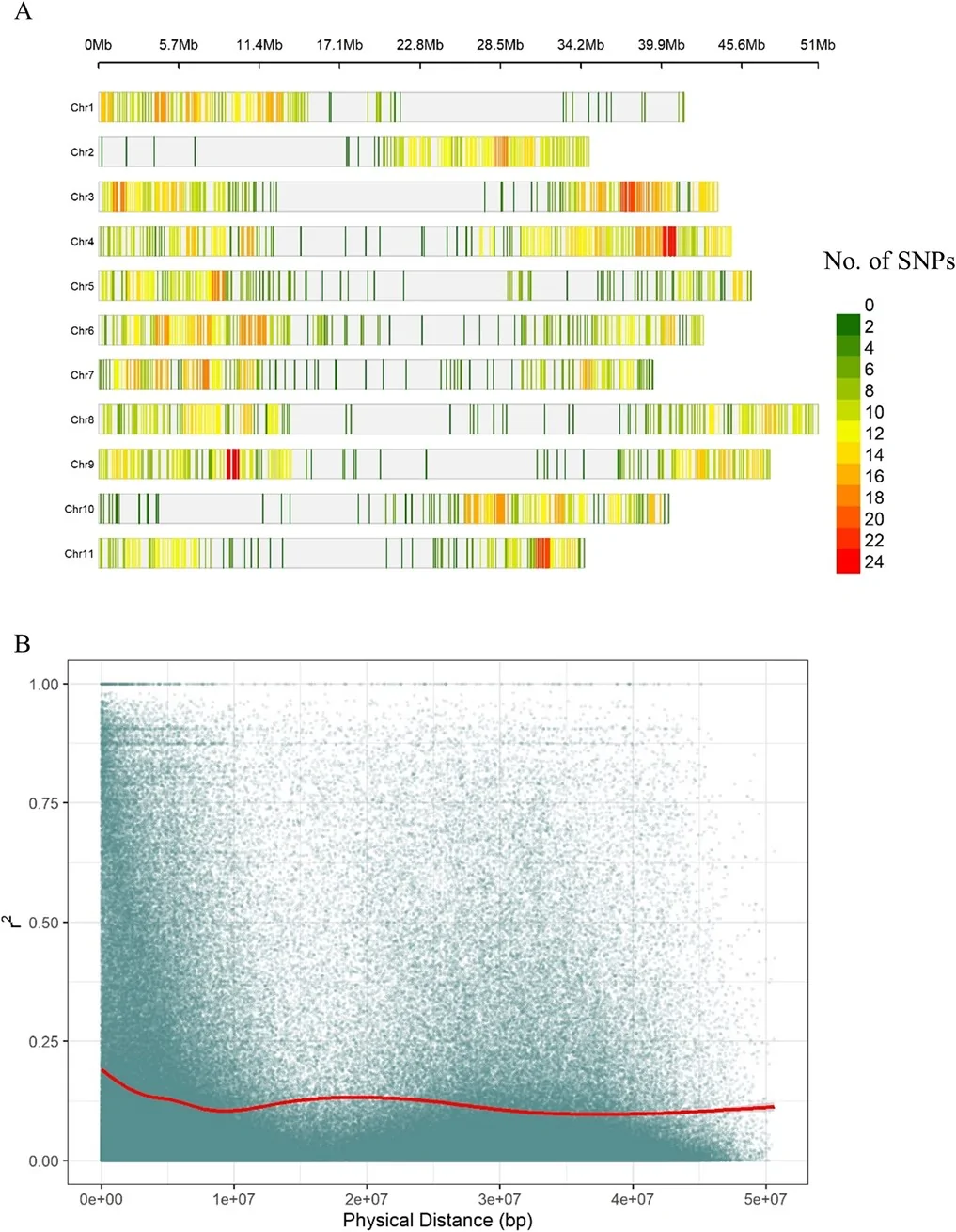
Distribution of 2792 SNP markers across the 11 banana chromosomes (A) and the genome-wide LD decay (B).

The number of SNP markers varied between 180 and 349 across all 11 chromosomes of the banana genome with an average of 254 markers (Fig. 1A). Chromosome 4 had the highest number of markers (12.5%), whereas chromosome 2 had the lowest number of markers (6.4%). Most of the markers are located at the distal part of the chromosome and rarely in the pericentromeric region. The LD decay analysis revealed moderate linkage disequilibrium with rapid decay within approximately 20 kb window (Fig. 1B). This pattern of LD decay could be explained by a high frequency of recombination in the distal part of the chromosomes leading to breaks of marker associations over genomic distance and the genetic diversity of the population analysed.

### Variance component estimates

**Figure 2:**
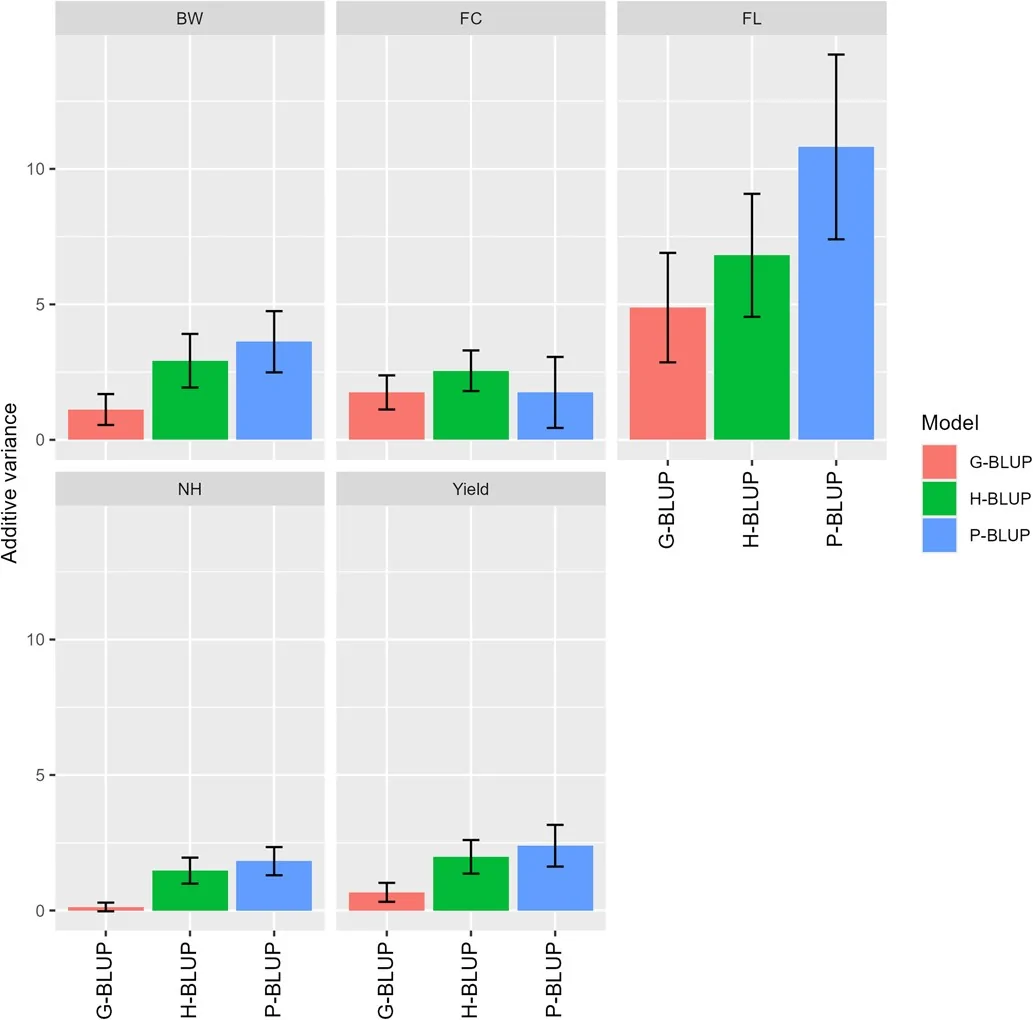
Additive variance components for the five yield-related traits estimated by the three models. BW in kg, FC in mm, FL in cm, yield in kg/year/plant.

The variance components, their standard errors (SEs), and likelihood ratio test results for the genetic and non-genetic effects as estimated by fitting the P-BLUP, G-BLUP, and H-BLUP models to all traits are given in Table 2, Tables S2 and S3. All three models revealed significant (*P ≤* 0.05) and highly significant (*P ≤* 0.001) additive effects for all yield-related traits and for plant height at flowering (PH), plant stature (PS), and plant height of tallest sucker (PHTS) agronomic traits. Significant (*P* ≤ 0.05) and highly significant (*P* ≤ 0.001) dominance effects for number of fruits (NF), fruit length (FL), and fruit finger weight (FW), and zero dominance effects for total number of suckers at flowering (NSF), number of standing functional leaves at flowering (NFL), days to flowering (DPF), and days from planting to harvest (DPH) were estimated by all three models. The G-BLUP and H-BLUP models estimated more dominance effects than the P-BLUP models. For all traits, the epistatic variance components estimated by all three models are close to zero except for PHTS that was significant (*P* ≤ 0.05) as estimated by the P-BLUP model, number of hands (NH) (*P* ≤ 0.001) as estimated by the G-BLUP and NF (*P* ≤ 0.001) as estimated by the H-BLUP. The strongest genetic variance component by all three models for most of the yield-related traits was the additive variance component, except NF under the P-BLUP method. The P-BLUP model estimated a larger additive variance component for all traits, than the H-BLUP and G-BLUP models (Fig. 2). The non-genetic variance components associated with the seasonal variation in planting time [year of planting (YP):season of planting (SP)] and the seasonal variation in flowering time [year of flowering (YF):season of flowering (SF)] were highly significant for most yield-related traits except for bunch weight at full maturity (BW) and NH by the H-BLUP model. The Plot, which represents the between plot variance for the fruit circumference (FC), FL, and FW traits was highly significant (*P ≤* 0.001) under all models.

**Table 2:** Variance components, their SEs and their significance based on a likelihood ratio test using the H-BLUP model.

Trait Category	Trait	Additive	Dominance	Epistasis	YF:SF	YP:SP	Plot	Residual
Yield	BW	2.92 ± 0.99^***^	0.00	0.00	0.24 ± 0.30	0.00	-	5.56 ± 0.58
	NH	1.47 ± 0.48^***^	0.08 ± 0.18^***^	0.00	0.19 ± 0.16	0.00	-	2.23 ± 0.24
	NF	1059.46 ± 1622.96^*^	650.29 ± 494.04^*^	1.32 ± 7.47^***^	1244.29 ± 792.45^**^	0.00	-	5578.80 ± 600.25
	FC	2.55 ± 0.75^***^	0.00	0.00	0.04 ± 0.14	4.56 ± 2.18^***^	4.73 ± 0.47^***^	0.28 ± 0.01
	FL	6.81 ± 2.27^***^	0.11 ± 0.77^***^	0.00	0.05 ± 0.37	3.26 ± 2.33^**^	16.02 ± 1.61^***^	1.64 ± 0.05
	FW	197.46 ± 59.05^***^	13.99 ± 24.59^***^	0.00	20.66 ± 24.11^***^	21.52 ± 29.69^***^	338.29 ± 34.38^***^	26.90 ± 0.75
	Yield	1.98 ± 0.62^***^	0.00	0.00	0.00	0.54 ± 1.04^***^	-	3.88 ± 0.42
Agronomic	NSF	0.29 ± 0.39	0.00	0.62 ± 0.39	1.29 ± 0.77^***^	0.17 ± 0.29	-	4.06 ± 0.42
	NFL	0.66 ± 0.34	0.00	0.00	0.67 ± 0.50^**^	0.38 ± 0.35	-	3.60 ± 0.38
	PH	284.69 ± 135.86^***^	36.31 ± 64.94^***^	0.00	273.81 ± 194.89	116.63 ± 119.03	-	1410.64 ± 147.09
	PG	6.82 ± 4.81	2.13 ± 2.94	0.00	2.40 ± 3.25	2.72 ± 3.40	-	51.02 ± 5.43
	PS	0.78 ± 0.45^***^	0.47 ± 0.36^*^	0.00	0.00	0.00	-	4.58 ± 0.47
	PHTS	349.98 ± 243.68^**^	0.00	0.00	232.61 ± 265.50^*^	147.42 ± 231.64	-	3024.96 ± 314.07
	NLTS	0.00	0.07 ± 0.15	0.01 ± 0.02	0.00	0.00	-	5.64 ± 0.52
Cycling	DPF	8816.49 ± 1689.24	0.00	0.00	823.45 ± 531.99^***^	2571.12 ± 1190.75^***^	-	1583.39 ± 173.03
	DFM	10450.48 ± 1945.51	45.56 ± 209.65^***^	0.00	9321.57 ± 3847.26	69.34 ± 112.74^***^	-	1137.44 ± 120.90
	DPH	10.68 ± 50.68^***^	0.00	0.35 ± 1.24	26690.71 ± 11065.07^***^	32270.99 ± 11976.72^***^	-	1299.74 ± 131.93

The ‘-’ means the term was not included in the model or SEs not estimated due to zero or near-zero estimates. BW in kg, FC in mm, FL in cm, FW in g, yield in kg/year/plant, PH in cm, PG in cm.

^*^ *P* ≤ 0.05.

^**^ *P* ≤ 0.01.

^***^ *P* ≤ 0.001.

The SEs for the variance components and the BLUP values of the traits assessed were generally lowest under H-BLUP, followed by the G-BLUP and highest under the P-BLUP (Table 2 and Tables S2–S6).

### Breeding values

The breeding values and the ranges, as indicated by BLUP values of the traits under the different models, significantly varied among the clones studied (Tables S4–S6). The P-BLUP model estimated higher BLUP values than the G-BLUP and H-BLUP, but the H-BLUP estimated higher BLUP values than the G-BLUP, for all traits. Noticeably, under the P-BLUP method, ‘Huti-White’ was the best-performing female parent, while ‘CV Rose’ was the best-performing male parent for the yield-related traits. However, 12 hybrids had BLUP values higher than the best male parent (‘CV Rose’) for all yield-related traits, with one hybrid (NM201901_ (153017C1/161564)_1) having twice the BLUP value of the best parent for NH. Ten of these hybrids came from crosses with ‘Huti-White’ as the female parent. However, using the G-BLUP model, ‘Pahang’ and ‘SH 3142’ were the best male parents while the female parents had similar performance for yield traits. Twenty-five hybrids performed better than the best-performing parent for yield traits. Eleven of these were the same as the best performers as identified by the P-BLUP method. Under the H-BLUP model, 43 hybrids performed better than the best parent for several yield-related traits. Four hybrids had the highest BLUP values for at least two yield-related traits, using all three models. Three of these four best-performing hybrids are from a cross with ‘Huti-White’ as the female parent (Tables S4–S5).

### Heritability and prediction accuracy of the models

The heritability estimates and prediction accuracies obtained from the P-BLUP, G-BLUP, and H-BLUP models are reported in Table 3. The median and mean values of *H*^2^ and *h*^2^ for the yield-related and agronomic traits under the H-BLUP model were greater than the other two models. Cycling trait DPF had the highest *H*^2^ under the P-BLUP method, but yield-related traits FL and FW had higher *H*^2^ under the H-BLUP method. The highest *h*^2^ was found for yield-related traits under the P-BLUP and G-BLUP models, and for DPF under the H-BLUP model. Heritability for DPH was estimated as zero by all three models. While the P-BLUP model estimated *h*^2^ > 0.50 for only three yield-related traits, the H-BLUP model estimated *h*^2^ > 0.50 for most yield traits, and the G-BLUP model had *h*^2^ > 0.30 for all yield traits.

**Table 3:** Estimates of broad-sense heritability (H^2^), narrow-sense heritability (h^2^), and prediction accuracy (ρ) using the three models.

			P-BLUP		G-BLUP			H-BLUP		
Trait category	Trait	*H* ^2^	*h* ^2^	*ρ*	*H* ^2^	*h* ^2^	*ρ*	*H* ^2^	*h* ^2^	*ρ*
	BW	0.64	0.64	0.75	0.42	0.28	0.49	0.58	0.58	0.76
	NH	0.66	0.66	0.77	0.51	0.07		0.62	0.59	0.77
Yield	NF	0.40	0.34	0.64	0.16	0.01		0.36	0.22	0.59
	FC	0.51	0.14	0.64	0.15	0.15	0.69	0.61	0.40	0.77
	FL	0.34	0.34	0.76	0.20	0.16	0.61	0.86	0.60	0.75
	FW	0.42	0.29	0.69	0.25	0.21	0.64	0.95	0.58	0.77
	Yield	0.54	0.54	0.75	0.43	0.27	0.45	0.52	0.52	0.76
	Median	0.51	0.34	0.75	0.25	0.16	0.61	0.61	0.58	0.76
	Mean	0.50	0.42	0.71	0.30	0.16	0.58	0.64	0.50	0.74
	NSF	0.34	0.34	0.70	0.20	0.00		0.24	0.08	0.44
	NFL	0.28	0.30	0.66	0.07	0.07		0.23	0.23	0.62
Agronomic	PH	0.32	0.30	0.66	0.24	0.18	0.39	0.27	0.24	0.62
	PG	0.37	0.33	0.62	0.20	0.01		0.29	0.22	0.57
	PS	0.56	0.37	0.60	0.25	0.12		0.45	0.28	0.59
	PHTS	0.20	0.00	0.00	0.22	0.19	0.41	0.20	0.20	0.56
	NLTS	0.12	0.11	0.45	0.18	0.03		0.04	0.00	0.00
	Median	0.32	0.30	0.62	0.20	0.07	0.40	0.24	0.22	0.57
	Mean	0.31	0.25	0.53	0.19	0.09	0.40	0.25	0.18	0.49
	DPF	0.68	0.36	0.71	0.00	0.00		0.69	0.69	0.91
Cycling	DFM	0.51	0.25	0.71	0.10	0.03		0.52	0.52	0.92
	DPH	0.00	0.00	0.37	0.00	0.00		0.00	0.00	0.22

BW in kg; FC in mm; FL in cm, FW in g; yield, yield/year/plant in kg; PH in cm; PG in cm.

In general, for yield-related traits, the H-BLUP model had higher heritability estimates and prediction accuracies than the other two models. While prediction accuracies (*ρ*) could not be estimated for NH, NF, NSF, NFL, plant girth at 100 cm from soil surface (PG), PS, number of standing functional leaves of tallest sucker (NLTS), and all cycling traits using the G-BLUP method, the H-BLUP method was able to provide estimates for these traits. For traits that could be predicted by all models, the P-BLUP model yielded better prediction accuracies than the G-BLUP method, whereas the H-BLUP method gave the highest prediction accuracies for yield-related traits and two cycling traits [DPF and days to fruit maturity (DFM)]. For agronomic traits, the P-BLUP model showed higher prediction accuracies compared to the other two models. The prediction accuracy (*ρ*) increased with heritability across all models.

## Discussion

Improving the efficiency of banana breeding requires accurate genetic evaluation of both breeding material and its progeny. Here, we assessed the use of the relationship matrix based on either pedigree or marker information and the combination of the two matrices in a diploid banana to partition genetic variance into the additive, dominance, and epistasis variances for various key traits. The study illustrated the different capabilities of the three models to account for confounding effects through their distinct ability to allocate genetic variance into its various components, with marker-based models being significantly more effective in accounting for non-additive variances.

### Heterozygosity and genetic diversity

The 2792 SNP markers selected in this study were informative, suggesting that they were suitable for reliable estimation of the genetic relationships between the genotypes and inference of genetic trait variation within the population [51, 52]. The markers had average MAF and PIC values within the range of the values reported in previous SNP-based genetic diversity research conducted on bananas [53–56], indicating that hybridization increases diversity in Mchare bananas, which are otherwise genetically identical. Conversely, low heterozygosity was observed for these markers and population, which is in line with the low genetic variation reported in cultivated bananas [5, 53, 57], and the classification of all Mchare in the same clone set [58]. This study also showed that 52% of the hybrids had Ho values above the mean Ho (0.13), with the top 10 hybrids coming from cross combinations with ‘Ijihu Inkundu’ and ‘Huti-White’ as the female parents. The findings indicate the presence of genetic variation and the potential usefulness of the studied genotypes for selection of hybrids and genetic improvement of the crop as heterozygosity and polymorphic information content have an influence on genetic variability and response to selection [59, 60].

### Performance of the models

#### Genetic parameters

The results indicated the existence of a large additive genetic variance component compared to dominance and epistasis, among the parents and clones for yield-related traits. The significant magnitude of additive genetic variation could improve breeding efficiency by identifying and selecting the best parents with high breeding values for desired complementary traits and generating new, improved breeding populations [29]. This could save time for the crops that are difficult to propagate, such as bananas and trees, where many clones are needed to capture all the genetic variance and enable selection within families or among clones [61, 62]. The four high-performing clones could undergo quality, disease, and pest resistance evaluation and be advanced as potential hybrids for release and/or for use in parental improvement strategies. The best-performing hybrids in this study were derived from crosses where ‘Huti-White’ was used as the female parent, an observation previously reported by Akech *et al.* [39]. In addition, non-additive effects for yield traits were found to represent only a small fraction of the total genetic variation and also observed by Akech *et al.* [39] in diploid Mchare clones and triploid plantains [63–65]. Consequently, narrow-sense heritability was equal to or nearly equal to broad-sense heritability for many traits. Traits with high (≥0.50) narrow-sense heritability estimates, such as most yield-related traits in this study, indicate strong potential for genetic improvement through direct selection [66, 67].

In this population, and for the traits evaluated, the additive variance and heritability estimate from models incorporating genomic relationship matrices (G and H matrices) showed reduced bias compared to the model that relied solely on pedigree information (A matrix). Although the P-BLUP model provided relatively accurate estimates of the additive variance component and heritability, as also observed in the study by Akech *et al.* [50], these estimates were slightly lower under the G-BLUP and H-BLUP models. This reduction may result from the more accurate variance partitioning capacity of the G and H matrices. When dominance and epistatic effects are present, part of the genetic variance is attributed to these components, thereby reducing the estimated additive variance and narrow-sense heritability [33]. In addition, the A matrix often makes approximations that can introduce substantial errors in reconstructing genetic relationships due to pedigree inaccuracies, affecting estimates of genetic parameters [33, 68]. Overall, the additive variance component and heritability estimates from the G-BLUP and H-BLUP models showed slightly lower SEs compared to estimates from the P-BLUP model. Among the three, the H-BLUP model yielded the smallest SEs for variance components, suggesting that it effectively partitioned variance into additive and non-additive components and provided more precise estimates of additive effects and narrow-sense heritability. The bias was larger in the P-BLUP model, probably due to the limited information of the A matrix compared to relationship matrices based on IBD. For example, in the P-BLUP method used in this study, pedigree information for the parents was unknown and thus not accounted for. In contrast, the G-BLUP and H-BLUP models can infer parental relationships from genetic marker data, with the H-BLUP model offering the added advantage of incorporating information from both genotyped and non-genotyped individuals [49]. The biased estimation of genetic components by pedigree-based methods, compared to genomic relationship methods, has also been reported in a clonal population of pine (*Pinus taeda*), where dominance effects for height were underestimated by approximately 50% using a pedigree-based model [33]. Similar observations have been reported in a simulation study of loblolly pine trees aimed at understanding the statistical properties of different models for estimating genetic variance components of complex traits [40, 69], as well as in a molecular and phenotypic profiling study of white Guinea yam (*Dioscorea rotundata*) breeding clones [29]. These results suggest that using genomic relationships estimated from molecular markers, instead of relying solely on pedigree information, increases the model’s ability to partition genetic variance more accurately. Lee *et al*. [70] also reported that the use of marker-derived relationship matrices alters the magnitude of variance components in additive genomic selection models. Further research has similarly indicated that genomic relationship matrices generally provide more accurate estimates of breeding values than pedigree-based matrices [43, 44, 71–73]. Reduced fertility in banana limits the number of available hybrids for evaluation and selection due to low seed set and germination rates [24]. Moreover, generating sufficient hybrid material for studies such as this requires several years [12]. These constraints explain the small sample size used to construct the genomic relationship model in this study. A small population size leads to a confounding effect, when a portion of non-additive variance is not distinct from additive variance, affecting estimation of genetic parameters, such as heritability. There will be less confounding with a larger sample size. Despite this limitation, this study provides an important foundation, establishing the principles and statistical models that can be further developed and applied to larger banana populations as they become available. In our study, combining both sources of information, pedigree from the A matrix and genetic markers from the G matrix, into a hybrid H matrix, significantly improved the accuracy of estimated genetic parameters.

#### Accuracy of genetic value predictions for key traits

Correlations between the true and EBVs based on additive variance were lower when using the P-BLUP model compared to the H-BLUP model. This could be explained by the H-BLUP model's more precise partitioning of additive and non-additive variance, which allocates a greater portion of the total variance to the dominance and interaction effects, thereby reducing the estimated additive variance. This suggests that pedigree-based models may overestimate additive effects, likely due to inflated additive variance estimates that capture non-additive components [33, 40]. Overestimation of breeding values can lead to inflated expectations of genetic gains, and inaccurate predictions of the genetic architecture of traits [74]. Another factor contributing to differences in prediction accuracies across methods may be the loss of individuals with missing genotypic data in the G-BLUP model. These individuals are dropped from the analysis, whereas the H-BLUP model includes them by using both their pedigree and genomic information. In this study, the prediction accuracies increased with higher narrow-sense heritability across all three methods, suggesting that narrow-sense heritability was the most critical factor influencing prediction accuracy, a finding also reported in white Guinea yam [29]. Since marker-based relationship matrices enhance the estimation of narrow-sense heritability [70], they contribute to more accurate prediction of progeny breeding values and, consequently, more precise estimation of genetic gains [69]. The superior performance of the H-BLUP model for yield-related and cycling traits, combined with the decreasing costs of genotyping, presents an opportunity to genotype a larger number of individuals in diploid banana breeding trials. With more individuals genotyped, the reliance on extensive phenotyping can be reduced by focusing on fewer individuals per family, thereby lowering costs and accelerating breeding efficiency and genetic gains. Moreover, in breeding programs lacking pedigree records, particularly when parental varieties are landraces as in this study, the H-BLUP method offers an advantage by inferring unknown pedigree relationships between and hybrids directly from genetic marker data. The superior performance of P-BLUP model in estimating higher prediction accuracies for agronomic traits suggests that additive genetic variation for the evaluated agronomic traits is largely structured by pedigree relationships within this F₁ population. This is also demonstrated by low narrow-sense heritability values estimated by the P-BLUP model, indicating strong environmental effects for agronomic traits. Consequently, molecular markers for agronomic traits provided limited additional information, and they do not substantially refine relationships beyond pedigree expectations.

## Conclusion

The opportunities to use genetic marker-based relationship matrices in addition to, or instead of, pedigree-based relationships have been made possible by the reduction in the costs of sequencing. This has consequently led to significant increase in the accuracy of breeding value predictions for progeny. The results presented in this study contribute to a better understanding of genetic variance component estimates, reliability, and prediction accuracy in banana, based on additive variance across three different models.

Using 2792 informative DArT-seq SNP markers, this study compares the prediction accuracies of methods that are based on genomic markers, and on only pedigree information, for several key traits in a diploid Mchare breeding population. The hybrid model (H-BLUP), which combines both pedigree and genomic relationship matrices, achieved the highest prediction accuracies for yield-related and cycling traits. In contrast, pedigree-only information (P-BLUP) showed the highest prediction accuracies for agronomic traits. These findings highlight the importance of incorporating genetic marker information over relying solely on pedigree or combined sources in the genetic analysis of yield-related traits, leading to more accurate selection decisions. In the diploid banana breeding program, the BLUP values estimated using the H-BLUP method can be applied to select clones either for advancement or release as products to farmers, as well as for identifying clones to be recycled as parents in recurrent selection for parental improvement. The selected clones for parental improvement can then be used for targeted pollinations, reducing costs and improving the efficiency of the diploid banana breeding pipeline.

The use of marker-based matrices enhances the accuracy of predicting progeny breeding values, thereby contributing to more precise estimates of genetic gain. This study also shows how the source of information used to construct kinship matrices affects overall genetic parameter estimates and prediction accuracy, paving the way for further refinement of statistical models in banana breeding programs.

## Materials and methods

### Experimental material and layout

Seventy-four Mchare banana hybrids were developed by hand pollination of four diploid female Mchare cultivars with pollen from five male parents in a factorial mating design resulting in 14 full-sib families (Table 4). The list of hybrids and their pedigree details used in this study is provided (Table S1). The ploidy level of the genotypes was determined by flow cytometry, using a ploidy analyser (CyFlow®-Sysmex, Software- CyViewTM 1.6, REF-CY-S-3039), following a standard protocol as described by Akech [75]. Hand pollination and subsequent evaluation of the study materials was carried out the International Institute of Tropical Agriculture (IITA), hosted at the Nelson Mandela African Institution of Science and Technology, Arusha, Tanzania (lat. 3°24′03′′S, long. 36°47′34′′E and 1200 m elevation), from 2020 to 2024. The site has a bimodal rainfall pattern with a rainfall average between 900 and 1400 mm year^−1^ [76]. The trial layout (comprising of the 74 hybrids and their nine parental genotypes), followed a randomized complete block design with three blocks, each genotype represented by two plants per block. Two ‘Matooke’ cultivars were planted around the experimental trial to counter the border effect.

**Table 4:** Factorial crossing design and the number of hybrids per full-sib family.

	Male (2*x*)					
Female Mchare landraces (2*x*)	Borneo (ITC0253)	Calcutta 4 (ITC0249)	CV Rose (ITC0712)	Pahang (ITC0609)	SH 3142	Total
Huti-White	9	11	10	10	2	42
Ijihu Inkundu (ITC1460)	4	2	3	6		15
Mchare laini	1	6	2	6		15
Nshonowa		2				2
Total	14	21	15	22	2	74

ITC, International Musa Germplasm Transit Center.

### Experimental management

Because the monthly rainfall amounts at the study site do not cover evapotranspiration rates [76], supplementary watering through drip irrigation (rate of 4 l hour^−1^) for 2 hours per day, 3 days a week was carried out to ensure each mat received at least 60 L per week. A mixture of mineral fertilizers and manure was applied on each mat. Mineral fertilizers, nitrogen, phosphorus, and potassium (NPK) were applied twice a year as 92 g urea, 100 g triple super phosphate (TSP) and 300 g muriate of potash (MOP) per mat. In addition, before the start of the rainy season, 20 kg of well-rotten farmyard cow manure was applied to each mat twice a year [76]. At flowering of the plant crop, one sucker (first ratoon) was allowed to grow while others were pruned (de-suckering). This was repeated at the time of flowering of the first, and second ratoon crops. Each banana mat was subsequently composed of three plants, i.e. the plant crop, the first ratoon and the second ratoon. De-suckering was performed monthly.

### Data collection

#### Phenotypic data

**Figure 3:**
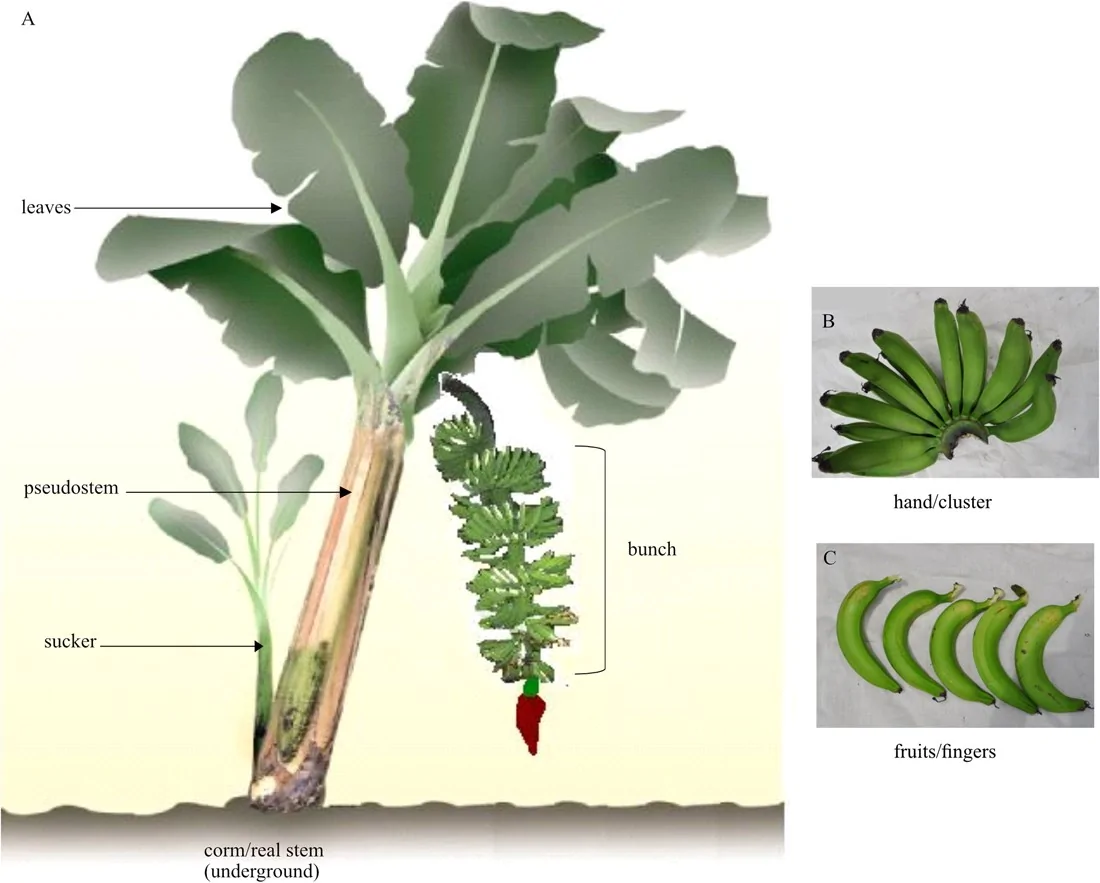
Phenotypic characteristics of a banana plant. (A) Schematic morphology. (B) The hand or cluster. (C) The fruits or fingers.

The phenotyping procedure and a detailed description of the traits analysed for this population follows the standard banana ontology [77] as described in Table 5, and a figure showing the major parts of the banana plant (Fig. 3) has been given as a guide. The data were collected for three years, from 2021 to 2024.

**Table 5:** Summary of traits considered in the study.

Trait category	Trait	Abbreviation	When and how measured
	Bunch weight (kg)	BW	At harvest, weight of the bunch at full maturity
	Number of fruit clusters in the bunch (count)	NH	Total count of number of clusters on the bunch at harvest
	Number of fingers in the bunch (count)	NF	Total count of number of fruits on the bunch at harvest
	Fruit external length (cm)	FL	At harvest, measured as the length of the external arc of a fruit, without pedicel
	Fruit circumference (cm)	FC	At harvest, measured midway the length of ten random fingers on a bunch
	Fruit weight (g)	FW	At harvest, measured as weight of each of ten random fingers from a bunch
Fruit yield	Yield per mat per year (kg)	Yield	A function of bunch weight, and crop cycle from planting to harvest of the plant crop
	Plant circumference at 100 cm from the collar (cm)	PG	At flowering, as girth of the pseudostem at 100 cm from soil surface
	Plant height (cm)	PH	At flowering, distance from the soil surface to the collar to the first leaves
	Plant stature (PG to PH ratio)	PS	Derived as a ratio (of PG/PH)
	Number of functional leaves (count)	NFL	At flowering, count of leaves that have 50% or more of their surface photosynthetic active tissue
	Plant height of tallest sucker (cm)	PHTS	At flowering, distance from the collar to the first leaves
	Number of leaves of tallest sucker (count)	NLTS	At flowering, count of leaves that have 50% or more of their surface photosynthetic active tissue
Agronomic	Number of suckers at flowering (count)	NSF	At flowering, total count of number of suckers
	Days from planting to flowering	DPF	Difference between flowering and planting date
Cycling	Days from flowering to harvesting	DFM	Difference between harvesting and flowering date
	Days from planting to harvest	DPH	Difference between harvesting and planting date

Yield per mat per year in kg was calculated using the following formula:


(1) \begin{equation*}\mathrm{Yield}\ \mathrm{per}\ \mathrm{mat}\ \mathrm{per}\ \mathrm{year}\ =\frac{\ \mathrm{Bunch}\ \mathrm{weight}\ \mathrm{per}\ \mathrm{plant}\kern0.5em \times 365}{\mathrm{Number}\ \mathrm{of}\ \mathrm{days}\ \mathrm{between}\ \mathrm{plant}\mathrm{ing}\ \mathrm{and}\ \mathrm{harvest}\ \mathrm{of}\ \mathrm{plant}\ \mathrm{crop}} \end{equation*}

#### Genotyping

Genomic deoxyribonucleic acid (gDNA) was obtained from 2 g of tender cigar leaf using the procedure described in a standard operating protocol adopted by IITA, using modified cetyltrimethylammonium bromide (CTAB) protocol according to Doyle [78]. Fifty μl of genomic DNA from each genotype at a concentration of 30 ng/μl were sent to Diversity Array Technology (DArT) Pty Ltd, Canberra, Australia, for sequencing (DArTseq). Genomic complexity reduction was done by digesting the DNA using a combination of *Pst*I and *Mse*I enzymes before library construction [79]. HiSeq 2500 (Illumina, USA) sequencer was then used to carry out the next-generation sequencing. The sequences in the fastq files were aligned to the fourth version of the double haploid (DH) Pahang banana reference genome [80] using a DArT in-house pipeline. The final SNP calls were allocated a value of ‘0’ if the genotype was homozygous for the reference allele, ‘1’ if the genotype was homozygous for the alternate allele and ‘2’ for the heterozygous genotype. While whole-genome sequence (WGS) would generate a larger number of variants, it would substantially increase sequencing costs, data storage requirements, and computational complexity without proportionally improving the performance of all models to address the study objectives. Therefore, a moderate number of genome-wide distributed SNP markers was considered a cost-effective and methodologically appropriate approach.

### Data analysis

#### Marker data

Data quality control and filtering were performed using the R package ‘dartR’ [81], with the summary statistics generated using the ‘*gl.report*’ function in ‘dartR’. Single-nucleotide polymorphism (SNP) markers with more than 80% of missing data, individuals with a call rate below 80%, loci with minor allele frequency (MAF) smaller than 0.05, monomorphic markers and loci with more than 25% heterozygosity were removed [53]. SNP-density plot was visualized using the R package ‘CMplot’. Pairwise LD between the filtered SNPs were determined using the squared coefficient of correlations between alleles (*r*^2^) with a 50-kb LD sliding window. LD decay plots of r^2^ against the physical distance in base pairs (bp) between SNPs from all chromosomes were generated using R package ‘ggplot2’. To properly visualize the LD decay trend, a smoothened LD curve generated from generalized additive model (GAM) implemented by geom_smooth option of ‘ggplot2’ was added to the LD plot.

#### Relationship matrices

The pedigree information of the 74 hybrids was used to construct the pedigree-based additive (A) relationship matrix using the ‘ainverse’ function in ASReml-R v4.2 [82] obtained by a copyright license, based on standard methods [35, 83]. This matrix represents the expected relationship coefficients derived solely from pedigree information [40].

The clonal model incorporating this A- matrix is hereafter referred to as the P-BLUP model.

In addition, a genomic additive numerator relationship matrix (A_G_) was calculated based on the final set of filtered good quality markers of 67 genotypes (59 hybrids and seven parents), following the formula described by VanRaden [44], as implemented in the ‘G.matrix’ function in the ‘ASRgenomics’ package, version 1.1.4 [49]. Accordingly,


(2) \begin{equation*} \mathrm{G}=\frac{{\mathrm{MM}}^{\prime }}{\sum 2 piqi} \end{equation*}

where G represents the genetic relationship matrix, M is an n × m matrix (n = number of genotypes, m = number of marker loci), which specifies the SNP genotype coefficients at each locus. The coefficients of the *i*th column in the M matrix are (0 − 2*p_i_*) for the genotypes homozygous for the reference allele (A_1_A_1_), (1− 2*p_i_*) for the heterozygous genotypes (A_1_A_2_), and (2 − 2*p_i_*) for the homozygous genotypes for the alternate allele (A_2_A_2_), where q_i_ and p_i_ are the frequencies of reference allele (A_1_) and alternate allele (A_2_) at locus i, respectively [44, 84].

The resulting A_G_ matrix contains the observed IBD proportions for each pair of individuals [85] The clonal model incorporating this A_G_ matrix is hereafter referred to as the G-BLUP model.

The third model employed used a combination of the A matrix and the A_G_ matrix, known as the H matrix. This H matrix has the same dimensions as the A matrix, and for the genotyped individuals, it can use either of their available relationship calculations, or a combination using the ‘H.matrix’ function in the ‘ASRgenomics’ package, version 1.1.4 [49]. The derivation of the H matrix is explained in detail by Gezan *et al*. [49]. The clonal model using the H matrix is hereafter referred to as the H-BLUP method.

#### Genetic analyses

Three alternative LMMs were independently fitted using either A, A_G_, or H matrices. The general structure of the LMM used for analysis using software ASReml-R v4.2 [82], is adopted from Nazarian & Gezan [40], and modified as follows


(3) \begin{equation*} \kern4.5em y=\mathrm{X}\mathrm{\beta} +{Z}_1a+{Z}_2 mf+{Z}_3 mf.c+{Z}_4b+\varepsilon \end{equation*}

where *y* refers to the vector of phenotypic observations, β is the vector of fixed effects such as block effects modelled as a conditional factor, and X is the associated design matrix. Z_1_ is the incidence matrix for additive effects, *a* ~ MVN (0, G) is the vector of random additive effects. G is the variance–covariance matrix of the additive effects corresponding to A${\sigma}_a^2$, ${A}_G{\sigma}_a^2$ and ${A}_H{\sigma}_a^2,$where A is pedigree-based, A_G_ and A_H_ are marker-based additive numerator relationship matrices, respectively, and ${\sigma}_a^2$ is the additive variance. Z_2_ is the incidence matrix for dominance or family effects, *mf* is the vector of the random male by female interaction or family effects, *mf* ~ MVN (0, I${\sigma}_{mf}^2$). Z_3_ is the incidence matrix for epistatic or clonal effects, *mf.c* is the vector of random clonal within family effects, *mf.c* ~ MVN (0, I${\sigma}_{mf.c}^2$). Z_4_ is the incidence matrix for the remaining random effects, *b* is the vector of the random effects such as seasonal effects, i.e. year and SP, and or flowering (YP, SP, and YF, SF, respectively), and plot effects (Plot) added only for FC, FL, and FW traits, ~ MVN (0, I${\sigma}_b^2$), and ε ~ MVN (0, I${\sigma}_e^2$) is the vector of independent and identically distributed residual errors [40].

Variance components were estimated using the restricted maximum likelihood (REML) method [86] and were used for obtaining estimates of the BLUPs for additive, dominance and clonal random effects. The estimated variance components, broad-sense (*H*^2^) and narrow-sense heritability (*h*^2^) [87] were estimated as:


(4) \begin{equation*} \kern2em {H}^2=\kern0.5em \frac{\sigma_g^2}{\sigma_{p\kern0.75em }^2}\kern1.75em \end{equation*}

and


(5) \begin{equation*} {h}^2=\kern0.5em \frac{\sigma_a^2}{\sigma_{p\kern0.75em }^2\ } \end{equation*}

where ${\sigma}_g^2$,${\sigma}_p^2$ and ${\sigma}_a^2$ is the total genetic, the total phenotypic, and the additive variance, respectively.

Heritability according to Cullis *et al*. [88] was used to compare the accuracy of the methods since it relies on the correlation between true and predicted genetic values, and not on the proportions of the variance components. We, therefore, estimated the reliability of the EBVs by the three models above as:


(6) \begin{equation*} {\rho}^2=1-\left(\frac{\mathrm{PEV}}{\sigma_{a\kern1em }^2}\right) \end{equation*}

where ${\rho}^2$ is reliability, ${\sigma}_{a\kern1em }^2$is the additive genetic variance and PEV is the prediction error variance or the average of the square of SE of the random additive effects or EBVs. The prediction accuracy (*ρ*) of each model for each trait was calculated using the formula:


(7) \begin{equation*} \rho =\sqrt{\rho^2} \end{equation*}

## Supplementary Material

Web_Material_uhag139

## Data Availability

The list of all plant entries, raw datasets and datasets generated during and or analysed during the current study referred to in the manuscript text as supplementary materials are publicly available in: Figshare: Integrating genomic additive relationship matrices improves the efficiency in diploid banana breeding. https://doi.org/10.6084/m9.figshare.29046386 [39]. The dataset has a CC-BY 4.0 license applied.
