# Supplementary material for: Integrating genomic additive relationship matrices improves the efficiency in diploid banana breeding
Source: Hortic Res. 2026 Apr 14;13(8):uhag139. doi: 10.1093/hr/uhag139 (PMC13563190; doi:10.1093/hr/uhag139)
Supplement: Supplementary file 1 [file web_material_uhag139.zip › Supplementary Tables S2-S3 .docx]

Table S2 Variance components, their standard errors and likelihood ratio test using the pedigree-based best linear unbiased prediction (P-BLUP) model

| Trait Category | Trait | Additive | Dominance | Epistasis | YF:SF | YP:SP | Plot | Residual |
| --- | --- | --- | --- | --- | --- | --- | --- | --- |
|  | BW | 3.62±1.13*** | 0.00 | 0.00 | 0.21±0.28*** | 0.00 | - | 5.5±0.59 |
|  | NH | 1.82±0.52*** | 0.00 | 0.00 | 0.19±0.16*** | 0.00 | - | 2.24±0.23 |
|  | NF | 1741.03±877.54*** | 343.55±445.35* | 1.45±8.03** | 1255.36±808.21** | 0.00 | - | 5473.72±59640 |
| Yield | FC | 1.75±1.31*** | 0.00 | 1.45±0.98 | 0.04±0.12*** | 4.71±2.23*** | 4.81±0.50*** | 0.26±0.01 |
|  | FL | 10.81±3.41*** | 0.00 | 0.00 | 0.05±0.41*** | 3.67±2.54*** | 16.83±1.75*** | 1.58±0.04 |
|  | FW | 202.08±151.11*** | 31.15±38.68*** | 61.32±90.59** | 15.38±21.37*** | 38.95±39.38*** | 341.50±35.64*** | 27.27±0.77 |
|  | Yield | 2.39±0.77*** | 0.00 | 0.00 | 0.01±0.10*** | 0.79±0.63*** | - | 3.81±0.42 |
|  | NSF | 1.37±0.61** | 0.00 | 0.00 | 1.21±0.74*** | 0.15±0.27* | - | 4.05±0.42 |
|  | NFL | 0.87±0.45* | 0.00 | 0.00 | 0.66±0.50** | 0.41±0.35* | - | 3.57±0.38 |
| Agronomic | PH | 397.46±204.95*** | 33.33±77.64 | 0.00 | 307.43±213.05 | 120.97±121.77* | - | 1436.21±153.14 |
|  | PG | 11.62±7.15* | 1.61±3.21 | 0.00 | 2.21±3.18 | 3.29±3.74** | - | 49.58±5.37 |
|  | PS | 1.24±0.68** | 0.64±0.50 | 0.00 | 0.00 | 0.00 | - | 4.50±0.48 |
|  | PHTS | 0.00 | 89.19±140.04* | 669.87±337.96* | 161.90±240.61* | 186.51±236.45* | - | 3039.60±316.51 |
|  | NLTS | 0.24±0.31* | 0.01±0.16 | 0.02±0.03 | 0.00 | 0.00 | - | 5.50±0.53 |
|  | DPF | 4464.86±2083.87*** | 0.00 | 3970.72±1700.18 | 826.99±531.26*** | 2590.38±1197.71*** | - | 1584.27±173.38 |
| Cycling | DFM | 4811.17±2155.59*** | 0.00 | 4965.96±1891.84 | 8957.33±3700.27 | 39.71±94.62*** | - | 1151.07±122.25 |
|  | DPH | 23.56±55.26 | 0.00 | 1.10±2.30 | 26898.28±10630.53*** | 32457.12±11594.41*** | - | 1279.57±129.32 |

The “-” means the term was not included in the model or standard errors not estimated due to zero or near-zero estimates. The *, **, *** indicate significant at p ≤ 0.05, p ≤ 0.01 and p ≤ 0.001, respectively. YF = year of flowering, SF = season of flowering, YP = year of planting, SP = season of planting, BW = bunch weight at full maturity (kg), NH = number of hands, NF = number of fruits, FC = fruit circumference (mm), FL = fruit length (cm), FW = fruit finger weight (grams), Yield = kg/year/plant, NSF = total number of suckers at flowering, NFL = number of standing functional leaves at flowering, PH = plant height at flowering (cm), PG = plant girth at 100 cm from soil surface (cm), PS = plant stature, PHTS= plant height of tallest sucker, NLTS= number of standing functional leaves of tallest sucker, DPF = days to flowering, DFM = days to fruit maturity and DPH = days from planting to harvest.

Table S3 Variance components, their standard errors and the likelihood ratio test using the genomic-based best linear unbiased prediction (g-BLUP) model

| Trait Category | Trait | Additive | Dominance | Epistasis | YF:SF | YP:SP | Plot | Residual |
| --- | --- | --- | --- | --- | --- | --- | --- | --- |
| Yield | BW | 1.12±0.57*** | 0.55±0.51*** | 0.00 | 0.19±0.31 | 0.00 | − | 6.27±0.72 |
|  | NH | 0.13±0.16*** | 0.18±0.23*** | 0.66±0.40*** | 0.11±0.16 | 0.00 | − | 2.45±0.29 |
|  | NF | 48.49±168.61* | 589.97±442.95* | 0.00 | 1303.59±898.48** | 0.00 | − | 5960.66±679.13 |
|  | FC | 1.75±0.63*** | 0.00 | 0.00 | 0.04±0.14 | 4.60±2.30*** | 5.06±0.58*** | 0.25±0.01 |
|  | FL | 4.88±2.02*** | 1.25±1.56*** | 0.00 | 0.00 | 6.37±3.97** | 17.85±2.03*** | 1.58±0.05 |
|  | FW | 113.68±42.92*** | 22.93±29.19*** | 0.00 | 34.04±34.20 | 0.00 | 374.55±41.79*** | 27.27±0.86 |
|  | Yield | 0.67±0.35*** | 0.38±0.35*** | 0.00 | 0.02±0.11 | 0.00 | − | 4.19±0.48 |
| Agronomic | NSF | 0.00 | 0.00 | 0.65±0.36 | 1.29±0.79*** | 0.00 | − | 3.88±0.43 |
|  | NFL | 0.20±0.18 | 0.00 | 0.00 | 1.03±0.74** | 0.34±0.36 | − | 3.64±0.43 |
|  | PH | 163.19±88.81*** | 45.76±76.39*** | 0.00 | 133.52±141.86 | 98.07±114.87 | − | 1326.74±153.87 |
|  | PG | 0.12±1.07 | 4.31±3.46 | 0.00 | 0.02±1.40 | 1.14±2.29 | − | 49.81±5.53 |
|  | PS | 0.24±0.23* | 0.28±0.30* | 0.00 | 0.01±0.11 | 0.07±0.18 | − | 4.55±0.52 |
|  | PHTS | 308.75±175.71** | 49.73±129.73** | 0.00 | 489.49±346.55* | 0.00 | − | 2371.27±276.85 |
|  | NLTS | 0.06±0.14 | 0.30±0.32 | 0.01±0.02 | 0.00 | 0.00 | − | 5.24±0.58 |
| Cycling | DPF | 0.00 | 0.00 | 0.09±0.46 | 22703.65±9772.8*** | 32062.85±11514.79*** | − | 423.58±45.10 |
|  | DFM | 4.35±7.34 | 8.36±13.60 | 0.28±0.68 | 21.02±24.75 | 0.00 | − | 291.81±31.11 |
|  | DPH | 5.81±17.14 | 0.00 | 1.06±2.23 | 17088.47±7796.75*** | 24321.54±9177.65*** | − | 863.52±97.17 |

The “-” means the term was not included in the model or standard errors not estimated due to zero or near-zero estimates. The *, **, *** indicate significant at p ≤ 0.05, p ≤ 0.01 and p ≤ 0.001, respectively. YF = year of flowering, SF = season of flowering, YP = year of planting, SP = season of planting, BW = bunch weight at full maturity (kg), NH = number of hands, NF = number of fruits, FC = fruit circumference (mm), FL = fruit length (cm), FW = fruit finger weight (grams), Yield = kg/year/plant, NSF = total number of suckers at flowering, NFL = number of standing functional leaves at flowering, PH = plant height at flowering (cm), PG = plant girth at 100 cm from soil surface (cm), PS = plant stature, PHTS= plant height of tallest sucker, NLTS= number of standing functional leaves of tallest sucker, DPF = days to flowering, DFM = days to fruit maturity and DPH = days from planting to harvest.
